# Barbed wire disease: British medical and lay prisoners of war and their perceptions of mental illness arising from captivity, 1939 to 1947

**DOI:** 10.1177/0957154X261427332

**Published:** 2026-03-06

**Authors:** Gabriel Lawson

**Affiliations:** 1 King’s College London, UK

**Keywords:** prisoners of war, mental health, captivity, diagnosis, neurosis

## Abstract

This article examines medical and lay perceptions of captivity-induced psychopathologies observed among British prisoners of war during the Second World War. Drawing on medical reports, POW memoirs, and camp publications, it explores how both medical and lay observers understood captivity-induced neurotic illness as a collective, environmentally driven disorder rather than an individual pathology. By reintroducing ‘barbed wire disease’ into the historical narrative, this paper challenges trauma-centred interpretations of military psychiatry and highlights the social and environmental dimensions of mental illness in captivity, offering new perspectives on rehabilitation, resilience, and the boundaries between mental health and ill-health.

## Introduction

From relatively early in the Second World War, an array of symptoms began to emerge in prisoners of war (POWs) held behind barbed wire. Most of these symptoms were psychological in nature, some were psychosomatic. They included feelings of depression and anxiety, insomnia, gastritis, aches and pains, social anxiety and other phobias, mood swings, and general irritability. Among British troops, this condition was variously termed ‘barbed wire disease’, ‘POW neurosis’, ‘gefangenitis’, or more colloquially ‘going round the bend’.

This article seeks to explore POWs’ own perceptions of this emergent malady, exploring the manner in which this unique condition was framed. While observers came to the problem from different perspectives (medical and lay, differing experiences of military service and varying knowledge of psychological theory) some common ground can be observed. POW syndromes were consistently portrayed in terms of environmental pressure, and seen as collective disorders resulting from the unique conditions of captivity *en masse*. Symptoms in POWs were also seen as the result of universal processes which impacted all prisoners to some degree regardless of background, rather than the result of constitutional defects which pre-dated the prisoner’s capture and only made themselves apparent during captivity. Fears of mental illness in prisoners of war were reliably framed in terms of prisoners’ return to civilian life, and fears for the wider national consequences of potentially long-term mental harm in former POWs. ‘Rehabilitation’ of some kind was consistently seen as necessary, in order to return prisoners to a normal psychological state and facilitate their re-entry to civilian society.

What is notable about this issue is its complete lack of a legacy. While ‘shell shock’ entered into the public’s lexicon and public memory of the First World War, ‘barbed wire disease’ currently possess no place in the popular image of the war and its aftermath. Partly this is due to trouble placing disorders limited by time and place within wider frameworks. The emergence of ‘shell shock’ and the supposed progression from physical to psychological aetiologies fits into a broader narrative which seeks to explain the rise of psychodynamic treatments and the emergence of modern psychiatry (Loughran, 2012). From this perspective, ‘POW neurosis’ is a blind alley – it struggles to be placed into wider narratives which link to modern conceptions of mental health. Ross (2019) in his work on accident neurosis posits that traumatic disorders with unclear aetiologies which do not lend themselves to a teleological interpretation of PTSD have been excluded from the historical narrative. This article extends that claim further, seeking to look beyond trauma as conceived by Ross and others. By writing ‘barbed wire disease’ back into the narrative, new possibilities begin to emerge. The conceptualisation of POW neurosis as shown by both medical and lay prisoners de-centralises trauma, and instead centres social environment. It de-centralises an individual’s constitution and pre-existing traits and instead centres ‘universal’ pressures which were seen apply equally to different individuals. It decentralises both physical and psychoanalytic forms of treatment in favour of relatively light-touch and socially-focused ‘rehabilitation’.

This article seeks to explore both medical and lay perspectives on captivity induced neurotic illness, while looking beyond the barbed wire to examine the wider frameworks in which this condition was created. ‘Barbed wire disease’ existed as a form of neurosis *sui generis*, produced regardless of a prisoner’s background and pre-existing personality, and grounded in the complex relationship between the individual and the collective. By including non-medical perspectives, this article hopes to develop a more rounded picture of the emergent illness, and one which touches on popular perceptions of psychological disorder. ‘Barbed wire disease’ has been used throughout as the earliest description to achieve prominence, but it must be stressed that the terminology describing the condition was always in flux, never successfully pinned down. The wide range of names used to describe the illness helps to demonstrate its protean nature.

This work primarily makes use of published commentaries on ‘barbed wire disease’ from medical and non-medical POWs, but also makes use of diaries, memoirs and camp newspapers to explore the widest possible range of perspectives on captivity-induced mental illness. Medically-trained prisoners of war were in the unique position of providing commentary on a phenomenon which they themselves were experiencing, and frequently texts from imprisoned medical officers blur the boundaries between the professional and the personal. Medical authors’ emotional states are often covered in some detail, even in medical journals where this sort of personal commentary was highly unusual.

This work does not attempt to cover the post-war programme of treatment for ‘unsettlement’ in former POWs at Civil Resettlement Units, which has been covered in detail elsewhere (Lawson, 2025; Makepeace, 2017). It focuses primarily on prisoners held in the European theatre. Where medical writing was published on Far East Prisoners of War (FEPOWs) after the war’s conclusion, the focus tended to be around reports of medical and surgical treatment delivered to prisoners rather than their mental state (Coates, 1946; Dunlop, 1946). The FEPOW experience was seen as radically different to that of European prisoners, in terms of the treatment received from captors, the day-to-day experience of captivity, and the support needed to recover after repatriation.

## POW syndromes

The history of psychiatry with relation to Second World War POWs has also been covered by a number of authors, particularly with regards to their resettlement in the war’s aftermath (Jones and Wessely, 2010; Lawson, 2025; Roberts-Pedersen, 2021). Makepeace’s (2017) notable *Captives of War* covers the psychology of POWs and their resettlement in some detail, as part of a broader exploration of the British POW experience.

More broadly the history of trauma, particularly that related to military service, has received a great deal of attention from historians (Leys, 2000) and scholars of the social sciences with an interest in the genealogy of traumatic disorders (Fassin and Rechtman, 2009; Young, 1995). In recent years, Ross’ (2019) work on accident neurosis seeks to broaden the study of traumatic disorders, shifting beyond what he sees as a linear path from ‘railway spine’ to ‘shell shock’ to PTSD, informed by a presentist assumptions. This article seeks to move even further, looking away from trauma and towards alternative explanations for what today would likely be seen as traumagenic psychological syndromes. As will be seen, POW observers largely neglected ideas of trauma, and instead focused on more subtle environmental influences, particularly the complex relationship between the individual and the group in a high-pressure environment.

Loughran’s (2008, 2009) work on ‘shell shock’ has covered in detail the complex roots of that condition and its relationship to psychological modernity. Loughran notes in her 2017 book on the subject that the act of bringing together a specific pattern of symptoms under a diagnostic label creates is a creative act. The creation of ‘shell shock’ as a diagnostic term made available ‘new forms of identification, understanding and behaviour’ (Loughran, 2017: 11). ‘Barbed wire disease’ was however a moving target, a disorder which was always in flux and never pinned down, hence the multitude of terms used for the condition. Commentators were unsure of whether it merited the term ‘neurosis’ or ‘disease’, in some cases actively avoiding this terminology and instead describing it as a ‘mental attitude’. Loughran’s (2012) article talks about plotting alternate routes from ‘shell shock’ to both PTSD and MTBI (mild traumatic brain injury). In this case, the plotting takes us off the map into an unexplored realm, where posttraumatic aetiologies for disorders were minimised in favour of psycho-social explanations which centred the collective rather than the individual.

This paper acknowledges the many lacunae surrounding the history of prisoners of war. The archetypal image of a POW is generally white, and as yet the role of ethnic minority POWs has been largely unaddressed.^1^ Between 15,000 and 17,000 Indian troops were taken captive in the Mediterranean Theatre, and around 70,000 were captured by the Imperial Japanese Army as it overran Malaya and Singapore (Khan, 2015: 76–78). While this is the largest cohort, other prisoners from across the Allied nations and their various territories and colonies were held by the Axis powers. The diversity of the POW population is clearly shown in the notes of POW psychiatrist Trevor Gibbens, described in detail later in this paper, who recorded treating West Indian, Chinese, Palestinian and Congolese troops within the German camp system (Gibbens, 1947). The section of this work exploring Gibbens’ treatment of Palestinian soldiers hopes in part to open up conversations about the diversity of the POW experience.

## Adolf Vischer and ‘barbed wire disease’

The concept of an emergent psychological illness occurring solely due to wartime captivity first gained widespread credence during the First World War. Early on, German psychiatrists claimed that mental disturbance among allied POWs was incredibly rare, partly due to the lack of stress from combat, and partly due to the lack of functional gain from displaying symptoms. However, as the war stretched on new viewpoints emerged. The term ‘barbed wire disease’ was coined in a camp newspaper for German internees in North Yorkshire to describe emerging symptoms, and by 1917 an Anglo-German conference was held at the Hague, where ‘Barbed Wire Disease’ was added to the list of conditions which could deem an individual worthy of repatriation (Makepeace, 2017: 155–156). Swiss surgeon and diplomat Adolf Vischer (1919) wrote a book with this title in 1918, translated into English the following year, which attempted to classify this newly observed condition and shed more light on its aetiology.

The symptoms associated with ‘barbed wire disease’ were vague – memory loss, lack of concentration, sudden bursts of anger and a definite ‘sensitiveness’. ‘Barbed wire disease’ in Vischer’s description is perhaps best viewed as a unique form of neurasthenia, with a definite symptomology developing over time, ‘recognisable in most of those who have lived for more than six months behind the barbed wire fencing’. Captivity was the cause, regardless of the circumstances of capture and the conditions of imprisonment: ‘the same objective influences operating upon different minds often produce psychical results of striking similarity’ (Bing and Vischer, 1919).

These objective influences were centred around the simultaneous loss of privacy and loss of sociality which occurred in POW and internment camps. In the *Lancet*’s review of his work, Vischer’s rationale for the emergence of ‘barbed wire disease’ was summed up in one sentence: ‘loss of liberty for an unknown period in close company with many others’ (*The Lancet*: Anon, 1918: 675). While solitary confinement was known to have adverse mental impact on inmates of civilian prisons, what occurred in POW camps was ‘confinement in mass’. Vischer (1919) was adamant that the material conditions prisoners were subject to had ‘but little influence on their mental condition’. Even in well-run camps in Switzerland, many cases emerged (p. 57). Symptoms were caused by the psychological and social impact of imprisonment away from one’s home and the strain of confinement, rather than the trauma of being taken captive or life in the camp. Vischer (1919) estimated that very few of those imprisoned for more than 6 months would be symptom-free, while out of a hut of thirty men, three would likely be regarded as ‘seriously affected’ by the others (p. 60). Worryingly, there appeared to be the possibility of permanent mental damage, as many prisoners ‘give one the impression of a personality that has been profoundly changed’ (Bing and Vischer, 1919: 697).

Vischer claimed ‘barbed wire disease’ to be a phenomenon as old as war itself, and gave examples of it from the diaries of Napoleon’s physician on St Helena. What made the condition visible in 1914–1918 was the sheer scale of captivity – Vischer estimated that between 4 and 5 million prisoners were held behind barbed wire. Many of these men would potentially develop a ‘damaged mentality’, with severe social consequences: ‘Europe will thus be infiltrated with individuals of abnormal psychical tendencies, who will not presumably be without influence on the collective psychology of the community’ (Vischer, 1919: 25). Vischer’s conception of ‘barbed wire disease’ resembles later definitions of ‘POW neurosis’ in terms of aetiology and potential social consequences, but lacks much of the psychological backing. Vischer, a surgeon by training, did not attempt to provide an in-depth psychological analysis of the cause or consequences of captivity neurosis, instead focusing primarily on symptoms and potential preventative measures. Vischer’s new diagnosis was however important in establishing the idea of captivity-induced mental illness, which lay dormant in the interwar period but was ready to be deployed after 1939.

## Context of British forces during the Second World War

More than 170,000 British troops were captured by German and Fascist Italian forces between 1939 and 1945. The vast majority of these men were taken during rapid Axis advances early in the war, in France, Greece and North Africa. To hold these men the Axis powers established POW camps throughout Germany, Italy and occupied Poland. Under the Geneva Convention, officers and enlisted men were held separately, with officers not required to work and enlisted men liable to be sent on working parties to local farms, factories or mines. Relations with German guards were generally frosty, although the Geneva Convention was generally obeyed (Makepeace, 2017: 64–68). By the end of the war, Germany had established 248 POW camps, 134 of which held British and American prisoners (Gilbert, 2006: 66). Conditions were basic, with prisoners held either in converted buildings (often a castle, a school or an orphanage) or in specially established hutted camps with barbed-wire fences. Prior to Fascist Italy’s surrender in 1943, Italian forces also held approximately 70,000 British and Commonwealth prisoners in 72 camps across Italy. While some of these men managed to escape and reach British lines in the chaos of Italy’s switching sides, many were rounded up by German forces and transported north for two further years of captivity. This sudden influx of prisoners to German camps made conditions there significantly worse due to overcrowding (Kochavi, 2005: 53–54). Food supplied by the German and Italian authorities was meagre, but prisoners in theory had access to regular Red Cross parcels which helped to supplement their rations. Distribution of these parcels seems to have been generally good, until Allied advances in the summer of 1944 threw the German transportation system into chaos (Makepeace, 2017: 185).

Prisoners were entitled to medical care, including psychiatric treatment, either from German medics or from captured British or Allied medical staff. While by 1945 the British Army had developed an intricate system of treatment for psychiatric conditions, frontline medics often lacked an in-depth knowledge of psychiatry, especially those mobilised and captured early in the war. The general curriculum for medical officers was light on psychological medicine, with an assumption that this would be a negligible aspect of medics’ workload. One POW doctor recalled that on his induction to the army in 1939, he was given only one lecture in military psychiatry as part of a curriculum focused primarily on field hygiene and treating battle wounds.^2^ In line with orthodox thinking about mental illness, it was assumed that many cases of neurosis were determined by an individual’s constitution and were therefore largely unpreventable and untreatable in the field. Even later in the war, when more attention was paid to ‘psychiatric casualties’, training materials stressed that these casualties appeared after ‘a long frightening battle’ featuring ‘little chance of sleep or rest’. Soldiers suffering from post-traumatic breakdown were ‘weary apathetic trembling slow-speaking men’ of previous good personality who were simply affected by ‘gross fatigue which unrelieved tension engenders’. These men were ‘true psychiatric casualties’, as opposed to ‘constitutional neurotics’ who were predisposed to mental illness before entering the army.^3^ As will be shown, ‘barbed wire disease’ added complexity into this by defying ideas of constitutional weakness and traumatic breakdown.

## George Collie: Rehabilitation and post-war problems

The earliest article on neurotic illness in POWs was published in June 1943 by a layman, Captain George Collie, who had escaped from German captivity the previous year (Collie, 1943). While Army psychiatrists had been pondering the issue of POWs since 1942, Collie’s article in *The Fortnightly* magazine kicked off a lively public debate over the correct treatment for repatriated prisoners. Public pressure, prompted in part by pieces such as Collie’s, was influential in pressuring the military authorities into action (Ahrenfeldt, 1958: 227–230). The advantage medically-trained commentators had over laymen like Collie was their use of medical terminology and their access to medical journals. Collie’s article in a popular periodical, while it did prompt public debate about POW resettlement, was ultimately eclipsed by published work from POW medical officers in defining ‘barbed wire disease’ for the authorities ultimately responsible for POW recovery and resettlement.

As a layman, Collie was nonspecific about the nature of the difficulties faced by returned POWs, simply claiming that anyone who spends time as a prisoner of war ‘becomes to a greater or less degree an abnormal person’. Without using any medical terminology, Collie (1943) claimed that the repatriated prisoner was ‘highly sensitive’ and needed delicate handling; any failures would impact ‘his outlook on life and consequently his ability to adjust himself to the changed conditions which he finds on his return to his country’ (p. 407). While lacking any formal psychological training or expertise, Collie possessed a great deal of faith in the ability of psychotherapeutic treatment to rectify any psychological and social issues experienced by prisoners. Collie’s plan for rehabilitation revolved around the establishment of ‘special rest centres’ for the treatment of prisoners, where on arrival a prisoner could be examined by a psychologist. In some cases, the prisoner would be declared healthy and released immediately, in most cases ‘treatment of some kind’ would be prescribed under a psychiatrist’s care. Collie claimed that in cases of ‘minor mental abnormality’ a psychiatrist could ‘by the use of the most simple equipment in a very short time effect a complete and lasting cure’. Prisoners would effectively be committed, as ‘no prisoner would be allowed to be discharged from the rest centre without the consent of the psychiatrist looking after his case’. Referring to a different form of expertise, Collie also suggested ‘the scientific placement in industry of those being discharged from the rest centres’, and if possible ‘the necessary training for placement in industry’. Personnel would need to be found to staff these rest centres, but luckily there was ‘a considerable and highly trained staff of Army psychiatrists who have been doing remarkable but little publicised work since the outbreak of the war’ (Collie, 1943: 409–411). Collie was presumably referring to the Department of Army Psychiatry, who did indeed find themselves responsible for the resettlement of British prisoners, and created a system of resettlement units for repatriated prisoners (Lawson, 2025).

Collie’s article foreshadowed the later discourse about POW resettlement by framing the issue in terms of citizenship and the deployment of scientific expertise. Collie was adamant that modern and scientific methods must be used to ensure POWs successfully returned to civilian life: ‘If the many thousand who are now in enemy hands are to make good future citizens, then for their own sakes as well as for the good of the community, it is important that the question of their treatment should be considered on scientific lines’ (Collie, 1943: 408). It is notable that Collie assumed that prisoners would have difficulty re-assuming their peacetime social roles in the home and the community, and that in order to help rectify this psychiatric expertise could be deployed to aid their resettlement. These two assumptions were to prove prophetic – Collie was not the only commentator who foresaw social problems arising from former POWs and who prescribed specialist psychiatric help as a solution to those problems. Collie was effectively suggesting using scientific expertise to solve the fundamentally social problem of POWs re-integrating with home communities.

The majority of the psychological and social issues identified by Collie in returned prisoners stemmed in his view from the emotional and physical distance captivity placed between POWs and their home communities. The prisoner behind the barbed wire ‘knows little of the Britain of 1943’ and the country which he has ‘spent long years in contemplating, and in anticipating pleasure in returning to, no longer exists’. Collie himself was nicknamed ‘Rip Van Winkle’ while in a military hospital due to his complete lack of knowledge about anything which occurred during his captivity. While in the rest centre the prisoner would be able to ‘rehabilitate his mind in knowledge of events which have happened during his captivity’ (Collie, 1943: 407–410). Collie was not alone in thinking of POWs as disordered in terms of a loss of connection with their home community. Within camp magazines, POWs frequently expressed fears that they were stranded in a state of isolation and stasis while their families and communities moved forward without them. *Westward Ho*, the magazine of the Devon and Cornwall club at Stalag IVB, noted that ‘For those amongst us imprisoned since the early days of the war the Motherland is going to seem very different. . . From the peaceful ‘nation of shopkeepers’ it was in 1938, England has, in this half-decade, become transformed into the most mighty war machine in the world’.^4^ While POWs expressed concerns that they were being left behind, those in European camps did manage to maintain contact with relatives through letters and possessed some awareness of what was occurring in Britain. For example, a study of prisoners’ letters reveals a sudden expression of panic after Germany developed the V1 bomb, with prisoners frantically writing home to establish their relatives had not been hit (Makepeace, 2013: 173). While *Westward Ho* bemoaned that POWs were at a distance from home, enough information was coming through to produce a double-page article on ‘Post-war Plymouth’, complete with a map of what the city would look like after redevelopment.^5^ What Collie’s analysis lacked in terms of medical terminology, it made up for in terms of piercing insight into the social nature of many repatriated prisoners’ problems.

## Philip Newman: Caisson disease and shell shock

The first major article on POW syndrome by an imprisoned medical officer was published late into the war by Major Phillip Harker Newman (1944). Prior to 1939, Newman was a surgeon at the Middlesex Hospital. He volunteered his services as soon as war broke out, and was captured outside of Dunkirk in the spring of 1940. After nearly 2 years of captivity, Newman escaped from his camp in northern France by hiding beneath the floorboards when the other prisoners were moved. He hid with a number of French civilians while travelling southwards, and was eventually repatriated via Gibraltar (Newman, 1983). In his article, Newman (1944) described POW syndrome as a form of ‘caisson disease’ (better known as ‘the bends’) as seen in deep-sea divers whose bodies ‘adapt themselves to an environment in which the atmospheric pressure is abnormally high’. Such men show no symptoms while in this environment, but if their return to the surface is too rapid symptoms can appear. The severity of symptoms depends partly upon the individual, but also on the length of time spent under water, and the pressure within the caisson.

The model of ‘Caisson disease’ is revealing. Compressed-air illness was investigated in the early 20th century by physician and self-experimenter John Scott Haldane (Boycott et al., 1908). Caisson disease was taken to be a predictable physiological response to placing the body under extreme pressure, which could only be prevented by slowly returning divers to the surface. This condition varied slightly depending on the individual subject to the pressure, but the effect was universal – any human being subject to such pressure would experience this illness to some degree. Newman therefore chose to use a physiological metaphor based around an illness caused by environmental pressure. While his article references only Adolf Vischer, Newman’s thought is at least indirectly influenced by Meyerian ideas of psycho-biology. Adolf Meyer conceived of neurotic behaviour as a ‘part-reaction’ to an individual’s environment, with the precise nature of the reaction determined by the individual’s circumstances, life-history and biology (Henderson and Gillespie, 1936: 419). Newman similarly claimed that psychological symptoms in prisoners represented the ‘response of a normal body to the process of recovery from exposure to an abnormal external environment’ which was ‘wrongly called a disease’ by uninformed observers (Newman, 1944: 8). To Newman, ‘POW neurosis’ did not exist, instead what was being seen was healthy men struggling to exit a difficult environment: ‘Barbed-wire disease is a misnomer. . . [it] is better termed a mental attitude’ which could be rectified through the correct form of treatment.

The emergence and progress of this ‘attitude’ was largely predictable. Newman created a schema in which ‘The Breaking-in Period’ immediately following capture is followed by a ‘Period of Convalescence’ and then a ‘Lengthy Period of Boredom’. Newman was adamant that POW syndromes should be seen as a normal, holistic reaction to the strains of camp-life rather than a form of neurosis. Any problems experienced by the prisoner on their return were in part social – the repatriate who returned home to find a sympathetic and understanding family could more easily ‘regain his emotional balance before he can again assume the responsibilities of his former life’. Prisoners lacking a ‘sound emotional foundation’ on their arrival would find a return to normality more difficult. Prisoners struggling to re-integrate might display a variety of vague symptoms such as irritability, phobias and social anxiety (as described by Vischer) but these should dissipate within 6 months to a year. Only a small number of cases which display exaggerated or persistent symptoms will require ‘individual treatment’.^6^ In terms of policy prescriptions, Newman recommended an ‘advice organisation’ be established for returned POWs ‘staffed by individuals, preferably medical practitioners, who understand prisoner-of-war life’. A central ‘rehabilitation centre. . . with consultant psychologists and a trained rehabilitation staff’ would also be needed for difficult cases (Newman, 1944: 11). Notably, Newman saw the need for specialist treatment for POWs, avoiding any mention of hospitalisation or orthodox psychiatric treatment.

Both Newman’s definition of ‘barbed wire disease’ as a phenomenon and his recommendations for treatment were to prove influential. Newman’s escape meant that his thoughts on repatriation were available before large numbers of POWs returned home, and his article was read eagerly by those planning POW resettlement. In the immediate aftermath of its publication, Newman’s article prompted a great deal of debate within the pages of the *British Medical Journal*. One commentator claimed gloomily that despite Newman’s portrayal of POW syndrome as natural and temporary ‘the very large majority of our returned prisoners of war will be problems for their lifetime’ (Harkness, 1944: 568). With this in mind, ex-POW clubs should not be formed as they will ‘retard rehabilitation’; instead POWs should be ‘encouraged to mix with others who have not suffered similar experiences and to merge as soon as possible into normal service or civil society’. A former POW wrote in to warn that prisoners held by the Japanese may well return ‘mentally and physically shattered’ and stated that repatriated POWs would require individual treatment and would not desire a ‘communal atmosphere’ of any kind (Vaughan Eley, 1944: 404).

Most notably, Newman’s writing attracted the attention of former shell shock specialist Millais Culpin. Culpin was a fierce advocate for psychodynamic therapies in response to war neurosis, both during the First and Second World Wars (Roberts-Pedersen, 2015). Despite Newman’s avoidance of the ‘witch word’ neurosis, Culpin argued forcefully in a reply that POW syndrome should be recognised as psychiatric in nature. Newman had argued against any ‘public acknowledgement of mental abnormality’ by providing POWs with psychotherapeutic treatment, but Culpin felt the correct solution was ‘to remove the stigma from “mental”’, not to deprive prisoners ‘of treatment that is now available for civilians at psychiatric clinics all over the country’. Culpin concluded his letter with the hope that Newman would ‘carry on the good work and place himself on the side of the psychiatrists, some of whom were struggling on behalf of their soldier patients a quarter of a century ago’ (Culpin, 1944: 158–159). Culpin appears to have predicted a similar trajectory for POW syndrome to that he claimed for shell shock – inaccurate somatic explanations and obfuscation eventually followed by the correct psychological interpretation and treatment. Within this narrative Culpin and his fellow shell shock doctors stood as progressive figures, slowly shedding light on a misunderstood condition while fighting public ignorance of mental illness. However, despite Culpin’s hopes, ‘barbed wire disease’ never gained the same unique status as shell shock.

## Archie Cochrane: Psychoanalysis and feigning illness

Dr Archibald Cochrane, best known for his later advocacy of randomised controlled trials and evidence-based medicine, also published an article on POW psychology based on his personal experiences in captivity (Cochrane, 1946). Writing up his notes in the immediate aftermath of the war, Cochrane separated the ‘Acute Gefangenitis’ (from the German word ‘gefangen’ meaning captured) experienced immediately after capture from the ‘Subacute’ and ‘Chronic’ forms experienced later on in captivity. Similar to Newman above, Cochrane did not think the syndrome he was describing was ‘any pathological condition’, rather a series of reactions to a difficult and unnatural environment. Cochrane (1989) had never worked in a psychiatric hospital, but hand undergone psychoanalysis with Freud’s disciple Theodor Reik in Berlin and Vienna during the early 1930s. Despite his familiarity with Freudianism, Cochrane’s article was largely free of psychoanalytic jargon and aimed squarely at the generalist medical practitioner. He did however choose to see the fundamental mechanism behind POW syndrome as ‘normal aggression being directed inwards’ producing in the short-term apathy and ‘symbolic death’ followed by ‘ill-directed aggression and escapist hysteria’. Without providing any further detail Cochrane (1946) speculated about the involvement of unconscious forces and ‘some unresolved residue of the Oedipus complex’ in producing the depressive symptoms associated with captivity (p. 283).

Cochrane described the period of time immediately after capture as one of shock and emotional collapse. Men who had previously been ‘well-disciplined soldiers’, capable of great exertions despite the lack of food and water, suddenly became ‘apathetic’, unable to move, unresponsive to orders and unwilling to escape despite obvious opportunities. The moment of capture often left a legacy within the prisoner’s psyche, something agreed by both POWs and outside observers. Many prisoners remarked on their unpreparedness for being taken captive, having given the issue little thought compared to being wounded or killed. Cochrane spoke to a large number of prisoners throughout his time in Germany, and found that ‘practically none of them even considered the possibility of being taken prisoner’. He himself ‘visualised death and wounds fairly often’ during the Battle of Crete but ‘never thought of being taken prisoner’. Cochrane (1946) suggested that ‘some strong unconscious factor’ prevented him thinking about being taken captive, despite the increasing odds of this occurring as the military situation worsened (p. 282).

Cochrane (1946) felt that when prisoners arrived at transit camps and settled down to some form of organised existence, symptoms of shock appeared to lessen, although a small group continued to suffer from ‘apathetic’ symptoms (p. 282). John Mustarde, a medical officer captured at Tobruk, claimed in his wartime memoir that those who tried to adapt to their new life immediately after capture were better served in the long-term. After being captured many prisoners began a conscious process of adjustment in order to ‘meet the long spell of empty existence which lay ahead’. However, some men ‘simply wandered about day after day doing nothing; making not even the slightest attempt to accept the situation and adjust themselves to the new life’. These men who failed to adapt would ‘show the scars of their experience in their minds and characters’ long after their return (Mustarde, 1944: 95). To both Cochrane and Mustarde, failure to adapt to a new community presaged long-term psychological damage.

Cochrane was intrigued by the boundary between physical illness, mental distress and pretence. Feigning illness, previously subject to punishment under King’s Regulations, suddenly became desirable as part of an effort to avoid work and thus hamper the German war effort. Cochrane admitted that he had to alter his ‘habitual severity’ with malingerers to suddenly adopt ‘an attitude of complacence if not actual encouragement’. Prisoners felt that malingering was their duty, and ‘this idea strongly reinforced any hypochondriac tendency’. Lines became blurred as ‘the differential diagnosis between psychoneurosis and malingering was very difficult’ (Cochrane, 1946: 283). POW camps appeared to exist as spaces where the line between mental health and ill health was thinner than elsewhere. As part of this, prisoners were aware of their status as potential lunatics and in many cases attempted to leverage this to gain better treatment. As some prisoners had realised in the Great War, within the confines of a POW camp, insanity could provide certain privileges, up to and including a ticket back home.^7^ Prisoners in some cases therefore sought to feign insanity as a form of protest or in order to inconvenience their captors without resorting to active sabotage. POWs held in Stalag 383 held a ‘Crazy Week’ to provoke the German authorities, in which they all pretended to hallucinate. The Camp Commandant eventually permitted walks outside of the barbed wire to head off what he feared was an epidemic of madness among the prisoners (McKibbin, 1947: 76). At Stalag Luft III, POW Paddy Byrne created a ‘Lunacy School’ where he instructed other prisoners on how to feign insanity in order to gain better conditions or repatriation (Gillies, 2011: 78).

However, leaning too far into the pretence of insanity seemed to carry real risks, as the line between fake and real madness appeared to be thin. In a memoir published after the war, J.E. Pryce (1958) recalled a friend who hatched a plan ‘to bluff the Germans into the belief that he was no longer responsible for his actions’. Within three weeks, ‘he really had lost his reason and had to be taken away in a strait-jacket’ (p. 188). Dr Trevor Gibbens, whose career is described below, felt that even in prisoners who were actively simulating symptoms there was often an underlying mental malady. Successfully simulating mental illness for a lengthy period of time would require ‘qualities of persistence and strength of character’ to a degree rarely found, and even in cases where malingering was definitely a factor this ‘does not exclude a mental illness’. Gibbens (1947) stated that the majority of British prisoners repatriated on the grounds of mental illness later claimed to have deceived the International Commission which adjudicated their cases, but he personally felt the commission was rarely deceived and the prisoners were actually mentally ill at the time of repatriation (pp. 122–129).

In hindsight, it seems as if many POWs were very willing to deem any unusual behaviour evidence of an oncoming breakdown. Prisoners, living under extreme stress and working under the assumption that some of their number would go ‘round the bend’, could spin any strange behaviour into evidence of lunacy. Gibbens noted that many prisoners lived in fear of slowly descending into a state of ‘stalag happiness’ which could only be cured by release. He recorded that one recently captured POW observed the long-term prisoners with fear, noting that they were ‘morose, warped in the use of English, and have fallen into a rut from which they endeavour to rise by an excessive amount of wishful thinking’ (Gibbens, 1947: 15). Guy Morgan, a journalist before the war captured in Yugoslavia, admitted in his memoirs that fears of insanity were common among POWs but felt that actual breakdowns were rare. Instead he claimed that widespread eccentric behaviour was common due to the ‘liberation of egos’ that captivity provided – he himself constructed a butterfly net and began to collect insects, which his camp-mates feared was a symptom of oncoming mental illness. Morgan (1945) claimed that the incidence of nervous breakdown was actually lower than in civil society, as a prisoner had no concerns about ‘his job, a roof over his head, food, rent, income-tax, or keeping up appearances’ (p. 134). To Morgan, captivity was not traumatic, but rather provided a degree of *liberation* from societal constraints which allowed POWs’ individual personalities to come to the fore. Again, the universal impact of captivity was held to act upon all prisoners to some extent – prisoners posited that that everyone was at risk of ‘stalag happiness’, rather than only a small number of constitutionally-impaired individuals.

## Trevor Gibbens: Psychotic and hysterical symptoms and the ‘constructive phase’

The most thorough analysis of the psychological impact of captivity from an imprisoned medical officer was created by Trevor Gibbens, later a noted forensic psychiatrist. Gibbens qualified in medicine at Cambridge before the war, and at the time of the Munich Crisis was working as a clinical assistant at the Maudsley. After joining the army, being captured in France in 1940 and attempting to escape from one camp, Gibbens was transferred to Stalag 344 (Lamsdorf), where he ran a psychiatric ward and outpatient clinic for POWs suffering from mental illness.^8^ Gibbens’ training at the Maudsley would have given him a good grounding in Meyerian psycho-biology, but he combined this with an early interest in psychoanalysis – he was inspired to become a psychiatrist while reading history at Cambridge, after encountering the works of Freud and Homer Lane (*British Medical Journal*: Anon, 1983). After his liberation, Gibbens treated a number of neurotic former POWs under military auspices at Larbert Rehabilitation Unit in Scotland. He also completed an MD Thesis based on his experiences, in which he compared convicted prisoners with the ‘normal healthy young men’ who enter captivity during wartime. In a criminal subject to frequent imprisonment ‘the inevitable effects of imprisonment are interwoven with his previous personality’, whereas POWs presented a control group where imprisonment without criminality can be observed (Gibbens, 1947: 1–3). The sizeable output of Gibbens’ writings on the subject (an MD Thesis of over a hundred pages) in addition to the sheer number of cases he examined allows a more in-depth exploration of POW psychology and emergent psychiatric illness.

Gibbens was effectively alone in attempting to integrate the literature on the psychology of POWs with that of prison psychology. Gibbens noted that work on the psychology of POWs almost never mentioned psychosis, while the literature on criminal prisoners almost never mentioned neurosis. Despite having little experience with convicted prisoners at this point, he felt that compared to POW neurosis ‘similar states occur in penitentiaries’, and prison psychoses as found in criminals could occur in POWs ‘rather less often, but to a considerable extent’ (Gibbens, 1947: 149–150).

Gibbens found the largest number of prison psychoses among the Palestinian Jewish labour companies captured in Greece and Crete. These companies were highly divided: members spoke different languages as they had emigrated to Palestine from across Europe and the Near East, and Gibbens felt that the groups contained both ‘honest and patriotic men’ and ‘shiftless individuals’. At the same time, the Palestinians were suspicious of other prisoners and fearful of the antisemitic German guards. Gibbens (1947) felt that their lack of ‘group ties’ made prison life especially difficult for this group, hence the large number of prisoners experiencing psychosis (p. 74). It may seem odd that the Nazi state placed these men in the POW camp system rather than concentration or death camps, but the fact that these were British army servicemen captured in uniform seems to have led the German military bureaucracy to treat them as British prisoners in possession of Geneva convention rights. Historians have generally characterised Nazi Germany’s policy towards British POWs as one of ‘reciprocity’: Nazi authorities generally treated British POWs well, subject to British authorities behaving in the same manner towards German prisoners. Exceptions to this were justified in terms of supposed poor conduct from the British side (Wylie, 2009).

Gibbens’ thesis does include post-traumatic cases, in the form of several patients suffering from hysterical symptoms, which he relieved through the use of hypnosis to reveal repressed memories. For example, one merchant sailor revealed when hypnotised that he had previous traumatic experiences of captivity from when he was held in the ship’s brig for violent behaviour. One patient had been suffering from hysterical blindness for 2 years, after lengthy hypnotic treatment he revealed that his condition had been triggered by the sight of his best friend burning to death inside a tank.^9^ This focus on hysteria and hypnotic treatments owed much to the legacy of shell shock. Gibbens saw captivity as being capable of exacerbating or causing psychological symptoms, but the treatments he utilised were also suitable for classical war neurosis.

In line with many experts on battle neurosis in the 1940s, Gibbens felt that a number of these patients were predisposed to neurotic breakdown and would have broken down on the battlefield had they not been captured. These patients experienced ‘a sense of relief from battle stress’ on entering captivity, and any neurotic symptoms already beginning to show from battle trauma disappeared. In the Stalag, such prisoners also underwent ‘a comforting loss of individuality in the herd’ which gave some protection against hysterical symptoms emerging (Gibbens, 1947: 66). Gibbens felt that pre-existing ‘schizoid’ and ‘obsessional’ personalities adapted to captivity very quickly – the former because they were freed from ‘the pressure of competitive life’, the latter because he ‘could regulate his life with great ease’. Chronic neurotics developed symptoms which produced some functional gain (such as removal from work parties, or better conditions in hospital) but did not remove them from the camp. Over time these cases realised that their only escape from prison life was ‘intrapsychic’ and began a slow descent into ‘the world of phantasy of the puerile or hysterical dementias’ (Gibbens, 1947: 69).

Beyond these relatively rare psychotic and hysterical states, Gibbens observed the emergence of neurotic symptoms in a much larger number of long-term POWs, in line with Vischer’s theories. This ‘barbed wire syndrome’ was spread throughout the camp ‘by group pressure’ as the entire community of prisoners slowly began to suffer from the strain of imprisonment (Gibbens, 1947: 24). In part this state was induced by the passage of time. For the prisoner, time passed differently to the free man – ‘the present passes with abnormal speed, but the past is left clearly visible, like foot-marks in a desert’. Gibbens felt that the symptoms shown by longstanding POWs were similar to the ‘Chronophobia’ seen in long-term criminal prisoners. This state occurs after settling into prison life – after a period of settling-in the prisoner is confronted with years of his sentence yet to be served (Gibbens, 1947: 28–36). Aside from prison psychiatry, there were also historical precedents for this. Gibbens claimed to have read about a ‘well known disease’ from the Middle Ages called ‘accidie’ which is best described as ‘part boredom, part depression’ and was common in monks who were given to the monastery at a young age.^10^

However, Gibbens disagreed with Vischer’s claim that ‘barbed wire disease’ emerges at 6 months and progresses steadily, instead claiming that between six and 18 months there is ‘a strongly beneficial and constructive phase’. Having surveyed 44 repatriates, 17 of the most recent captives reported positive aspects to internment. After this constructive phase ends, there was a ‘crisis’ stage marked by increasing irritability and psychosomatic symptoms. Gibbens himself managed to avoid this by moving between several hospitals and therefore never developing an unchanging routine. Once again using a monastic metaphor, Gibbens claimed that the beneficial stage is ‘the equivalent of the virtues found in monasteries, or for laymen who go into retreat’.^11^ In medical terms, the period of personal rumination and inner growth experienced by prisoners resembled ‘the isolation treatment of the chronic alcoholic. . . the psychological aspects of the Weir Mitchell treatment, and of the present penal system’ (Gibbens, 1947: 26). The ‘Weir Mitchell treatment’ Gibbens refers to is perhaps better known as the ‘rest cure’, a late 19th century treatment for neurasthenia and nervous illness, primarily prescribed to female patients (Martin, 2007). By the 1940s, the rest cure had entered into popular culture but had no firm place within medical treatment (Stiles, 2012). In reaching for three disparate examples, including one from decades prior to his writing, Gibbens was struggling to place the ‘constructive phase’ within contemporary psychiatry. Much like Newman and Cochrane, Gibbens framed prisoners’ reactions in terms of environment and social change rather than pathology. Unlike these two authors, Gibbens also attempted to explain these changes in psychoanalytic terms. Gibbens (1947) felt that the recovery phase experienced by prisoners was due to the re-repression of combatant aggression, which led to a re-assertion of the super-ego, while the libido was forced into ‘unusual psychic paths’ (p. 27).

Other prisoners frequently described this stage in memoirs or letters to family. John Chew, captured in France in 1940, wrote in a letter to his parents that: ‘One golden opportunity is offered to all us prisoners, the ability to reflect on past life from a distance, to see mistakes and weaknesses and on return to have perhaps the only chance to replan and start life in certain spheres’.^12^ In line with Gibbens’ theory on the constructive phase, at this point Chew had been prisoner for just short of a year. Mustarde (1944) linked this feeling of introspection and growth to a liberation from the ‘humdrum, suburban lives’ prisoners had previously lived. Having lost ‘every vestige of our former selves’, POWs found themselves in ‘a unique position from which to study ourselves. . . in an almost detached fashion’ (p. 129).

As part of his investigation of the psychological impact of captivity, Gibbens was interested in the relationship between individual and collective needs in the camp, and where these two forces clashed. For him, captivity appeared to strip away the veneer of civilised behaviour and reveal more primal instincts underneath. Gibbens challenged a number of prisoners to write him essays on captivity, which revealed a startling ‘lack of comradeship’ and resentment towards the crowd of fellow inmates. One prisoner wrote ‘the wheel of life has thrown me into the company of men who have taught me that familiarity breeds contempt’. Another wrote that ‘characters which in civil life may have seemed good have suffered a very obvious analysing and in many cases have been rendered down to show the real type, hidden beneath a polished surface’. A third prisoner claimed ‘what we see here can only be a reflection of the state of the great community outside, and I have arrived at this conclusion. . . we humans are all basically, utterly selfish’ (Gibbens, 1947: 13–14). The ‘Barbed wire syndrome’ was to some extent the result of a disillusionment with the collective, as the more unpleasant traits of individual characters came to the fore.

The only forms of group expression Gibbens could observe in prisoners was exercised in terms of fervent loyalty to the flag and to one’s comrades. Beyond this ‘the prison camp is a collection of warring individuals, for it is a functionless society’. Any altruistic activities were assumed to be attempts to pass the time, and communal cooking and eat was doomed due to mutual suspicion of being cheated. Group feeling was only strong ‘where there remains the residue of a military function – escaping parties, regimental teams etc.’ Gibbens noted that discipline among POW medical orderlies he supervised was excellent, ‘in spite of the absence of punishment for default’. The lack of purpose meant that collective life was unable to properly form, hence Gibbens’ claim that the ‘isolation of the individual in a functionless society’ was vital to understanding POW psychology. The only non-military groups were ‘remnants of pre-captivity forms – professional or minor social groups held together in mutual defence against the intrusion of the herd’ (Gibbens, 1947: 21–22). Several prisoners claimed that conditions of camp life had made them immune to herd mentality. Morgan (1945) suggested that the ‘peculiarly individualistic character of prisoner-of-war life’ rendered prisoners ‘immune to the normal promptings of herd mentality and mass enthusiasm’ and led to a scepticism ‘towards slogans, theories and ideas’ (p. 130). Use of the ‘herd’ in this context likely refers to the work of Wilfred Trotter, a notable theorist of group behaviour. A surgeon by training, Trotter published his highly influential *Instincts of the Herd in Peace and War* in 1916, which raised several concerns surrounding human nature, politics and war (Swanson, 2014). Trotter disagreed with Freud’s account of repression, and theorised the existence of an associational impulse, an inbuilt ‘sensitiveness’ to herd opinion. In Gibbens’ description, POW life is complicated by a simultaneous attraction to and repulsion from the herd. In Morgan’s, the reality of being held in close proximity to other for lengthy periods of time paradoxically provides protection against the herd and encourages individualism.

Gibbens’ thesis is also useful due to the glimpses it provides into prisoners’ assessment of their own mental state. Gibbens noted that depression was rarely reported as prisoners ‘thought it too normal to constitute an illness’. Men of ‘good personality and morale’ reluctantly reported sick with psychosomatic anxiety symptoms but on examination were often severely depressed (Gibbens, 1947: 56). Individual cases described by Gibbens provide more detailed stories. A New Zealand fighter pilot developed bizarre sexual symptoms (spontaneous orgasms) in captivity and reported to Gibbens that he felt he had a ‘double personality’. He later said that he thought he had ‘strained the nerve of erection by masturbation’ and that owing to this he feared that his children would be deformed. Another pilot officer treated by Gibbens confided in him after the war that he had been ‘helping in the tunnel in Luft III from where the 70 odd officers escaped, 50 being shot’ (an incident later termed ‘the Great Escape’). This occurred several weeks before the pilot’s psychotic breakdown and ‘I believe affected me so much as to hasten my illness’ (Gibbens, 1947: 53; 83). Prisoners’ ideas of how the mind functioned and how captivity could impact it were often highly idiosyncratic, but provide brief glimpses into popular ideas of the aetiology of mental illness in this period.

Gibbens’ pilot with sexual symptoms raises a topic notable by its conspicuous absence – masculinity. POW camps were all-male environments where gender was often complicated and sexuality occasionally fluid (Makepeace, 2018). While both the prisoners and Gibbens himself never make masculinity explicit, it lurks in the background of medical and lay attitudes to psychological symptoms. Gibbens’ framing of ‘good personality and morale’ was to some extent grounded in tropes about normative masculinity, and to some extent prisoners exhibiting symptoms were deviating from this norm. The fact that symptoms were presented as a natural and inevitable reaction to unpleasant and unusual circumstances likely made them easier to process for doctors and laymen.

## Conclusion

While it left remarkably little in terms of public legacy, especially when compared to shell shock, ‘barbed wire disease’ represents an interesting case study. It presents an example of a non-traumatic aetiology for neurotic illness when much attention was focused on battlefield breakdowns. It existed as a collective pathology, stemming in some way from the complex relationship between the individual and the community. Treatment was seen in terms of ‘rehabilitation’, often on collective lines, with little to no focus on orthodox psychiatric treatments.

In order to explain this complex illness, POW observers, both medical and lay, took inspiration from varied and sometimes unusual directions. This includes Caisson Disease, Silas Weir Mitchell’s ‘rest cure’ and medieval monks. Lacking clear explanations for ‘barbed wire disease’, commentators groped for potential inspirations elsewhere. What is clear in hindsight is the manner in which these inspirations differed from ‘classical’ ideas of war neurosis and post-traumatic illnesses. Psychopathologies in POWs were viewed as both collective and environmentally driven in a way in which other conditions were not. ‘Barbed wire disease’ could be seen as an illness related to time (as in the work of Trevor Gibbens) and related to distance (as in Collie’s article).

The shift towards a more environmentally-focused model of causality allowed POW neurosis to be seen as a potentially temporary condition, reversable through a spell in a therapeutic environment or some other light-touch form of treatment. This had the dual advantage of avoiding stigmatisation among repatriated POWs experiencing symptoms, and avoiding potential long-term medical and financial obligations for the military authorities. All parties benefited from this view of captivity-induced illness: former POWs could avoid the stigma of being diagnosed with a mental illness; military authorities could avoid accepting responsibility for any long-term consequences arising from the captivity POWs had been subject to.

What is also clear is that non-medical prisoners took an active interest in psychopathologies arising from captivity and sought to explore and explain them. White (2016) has claimed that POWs in this period were viewed in psychological terms by lay and medical observers, but this was not simply ‘a case of scientific ideas being watered down for the public; the ideas were originally expressed by non-specialists in letters and popular periodicals before being taken up by the psychiatrists’ (p. 154). George Collie stands as the best example of this phenomenon, but many other prisoners sought to use what knowledge they had to explore the psychological impact of captivity, regardless of whether they were medically qualified.

## Notes

1. Romain’s (2017) *Race, Sexuality and Identity in Britain and Jamaica: The Biography of Patrick Nelson* covers Nelson’s capture and some of the issues black POWs faced while in German captivity.

2. T.C.N. Gibbens, ‘Captivity’ unpublished memoir, 1990, Museum of Military Medicine, Aldershot, RAMC/PE/1/308/GIBB, p. 3.

3. ‘Field Psychiatry for Medical Officers’, April 1944, Museum of Military Medicine, Aldershot, MO 84 Box 382.

4. Stalag IVB Camp Magazine, *Westward Ho*, August 1944, Vol. 1, No. 1, Imperial War Museum, London, Box No. Misc 72(1086), p. 5.

5. Stalag IVB Camp Magazine, *Westward Ho*, p. 5.

6. Department of Army Psychiatry Technical Memorandum No. 13, ‘The Prisoner of War Comes Home’, May 1944, Wellcome Library, London, SA/TIH/B/2/1/2/2/1, p. 9.

7. Notably E.H. Jones successfully deceived his Ottoman captors into believing he was insane and received an early release on compassionate grounds, see Jones (1930).

8. Gibbens, ‘Captivity’, p. 74.

9. Gibbens, ‘Captivity’, pp. 77–8.

10. Gibbens, ‘Captivity’, p. 101.

11. Gibbens, ‘Captivity’, p. 102.

12. J. Chew, Letter to Mr and Mrs H.E. Chew, 2/4/41, Imperial War Museum, London, Documents.566, p. 1.
